# The Role of Textured Material in Supporting Perceptual-Motor Functions

**DOI:** 10.1371/journal.pone.0060349

**Published:** 2013-04-02

**Authors:** Dominic Orth, Keith Davids, Jon Wheat, Ludovic Seifert, Jarmo Liukkonen, Timo Jaakkola, Derek Ashford, Graham Kerr

**Affiliations:** 1 Centre d’Etude des Transformations des Activités Physiques et Sportives (CETAPS) - EA 3832, Faculty of Sports Sciences, University of Rouen, Rouen, Haute-Normandie, France; 2 School of Exercise & Nutrition Sciences, Queensland University of Technology, Brisbane, Queensland, Australia; 3 FiDiPro Programme, Faculty of Sport and Health Science, University of Jyväskylä, Jyväskylä, Central Finland, Finland; 4 Centre for Sports Engineering Research, Sheffield Hallam University, Sheffield, South Yorkshire, United Kingdom; 5 Institute for Biophysical and Clinical Research into Human Movement, The Manchester Metropolitan University, Alsager, North West England, United Kingdom; 6 Movement Neuroscience Program, Institute of Health & Biomedical Innovation, Queensland University of Technology, Brisbane, Queensland, Australia; CSIC-Univ Miguel Hernandez, Spain

## Abstract

Simple deformation of the skin surface with textured materials can improve human perceptual-motor performance. The implications of these findings are inexpensive, adaptable and easily integrated clothing, equipment and tools for improving perceptual-motor functionality. However, some clarification is needed because mixed results have been reported in the literature, highlighting positive, absent and/or negative effects of added texture on measures of perceptual-motor performance. Therefore the aim of this study was to evaluate the efficacy of textured materials for enhancing perceptual-motor functionality. The systematic review uncovered two variables suitable for sub-group analysis within and between studies: participant age (groupings were 18–51 years and 64.7–79.4 years) and experimental task (upright balance and walking). Evaluation of studies that observed texture effects during upright balance tasks, uncovered two additional candidate sub-groups for future work: vision (eyes open and eyes closed) and stability (stable and unstable). Meta-analysis (random effects) revealed that young participants improve performance by a small to moderate amount in upright balance tasks with added texture (SMD = 0.28, 95%CI = 0.46–0.09, Z = 2.99, P = 0.001; Tau^2^ = 0.02; Chi^2^ = 9.87, df = 6, P = 0.13; I^2^ = 39.22). Significant heterogeneity was found in, the overall effect of texture: Tau^2^ = 0.13; Chi^2^ = 130.71, df = 26, P<0.0001; I^2^ = 85.98%, pooled samples in upright balance tasks: Tau^2^ = 0.09; Chi^2^ = 101.57, df = 13, P<0.001; I^2^ = 72.67%, and in elderly in upright balance tasks: Tau^2^ = 0.16; Chi^2^ = 39.42, df = 5, P<0.001; I^2^ = 83.05%. No effect was shown for walking tasks: Tau^2^ = 0.00; Chi^2^ = 3.45, df = 4, P = 0.27, I^2^ = 22.99%. Data provides unequivocal support for utilizing textured materials in young healthy populations for improving perceptual-motor performance. Future research is needed in young healthy populations under conditions where visual and proprioceptive information is challenged, as in high-speed movements, or where use of equipment mediates the performer-environment interaction or where dysfunctional information sources ‘compete’ for attention. In elderly and ailing populations data suggests further research is required to better understand contexts where texture can facilitate improved perceptual-motor performance.

## Introduction

The somatosensory system in humans includes many sensory components such as plantar cutaneous mechanoreceptors, joint receptors and muscle receptors. Previous research has shown that the stimulation of sensory receptors in the skin via simple mechanical deformation of the surface by added texture (e.g., addition of nodules and protuberances on the surface of a shoe insole or a standing area) can improve perceptual-motor system functionality in upright balance [1]. These findings have since been replicated, under similar experimental task constraints, in a number of other samples of healthy, young participants [2]–[6]. The morphology of textured surfaces used in previous work has been defined by a huge parameter space including variables such as nodule height, shape, material, area and packing density. Research has evaluated the efficacy of added texture that passively deforms the plantar surface of the foot in a variety of populations (elderly [2], [3], [5]–[12] and those with ailment [9]–[11], [13]–[15]) and under a range of perceptual-motor task constraints, including upright balance [1]–[7], [12], [15]–[18], walking [8]–[11], [13], [14], [18]–[22] and joint position discrimination [23], [24].

### Somatosensory Regulation of Movement and Effects of Stimulating Cutaneous Receptors with Added Texture

The somatosensory system has functionally distinct and interacting roles in the nervous system [25]. Structurally it pervades both central and peripheral regions through which it functions to convey information to the brain from the skin [26], [27] and musculoskeletal [28] system regarding weight bearing activities and the relative positioning of body parts [25]. Information, much of which is induced by movement [29], [30], ascends to the brain via somatosensory tracts [25] where it may be perceived or integrated with visual and vestibular inputs [31] at sub-conscious [32], [33] and conscious [34]–[36] levels. These multilevel interactions [37] between the somatosensory and other nervous system sub-components reflect its complex role in supporting action [38]–[40].

Previous research has reported that textured materials can influence somatosensory system functioning during mechanical interactions with specialized cutaneous receptors [41]. Cutaneous end organ afferents are specialized to be preferentially sensitive to specific spatio-temporal scales of mechanical energy [41], [42], meaning that acceleration, velocity and intensity of action can be discriminated. It appears that all of these characteristics might be stimulated by added texture [41].

Research to date has sought to ascertain effects on perceptual-motor performance of added texture at the plantar surface of the foot sole under various task constraints (i.e. static and dynamic balancing). Varied effects have been observed in studies which have added texture to articulation surfaces and determining the contexts in which textured materials provide the greatest benefits for perceptual-motor functionality is clearly an important research task. This is especially the case in populations, such as those with ageing [43] or diseased nervous systems and those with a high falls risk [44], where individual and social benefits are palpable (for reviews see [40], [45]–[48]). Evidence suggests that textured material can ameliorate reduced perceptual-motor system functionality related to ageing [2], [3], [5]–[7], [9], [11], disease [10], [11], [13], [14], and previous injury [15], and that observed benefits are not lost over extended time scales due to habituation to stimuli [2], [3], [11], [13].

### Textured Material Interventions

In contrast to growing evidence of improved perceptual-motor performance with added texture, a significant body of work has also shown no significant effects of texture on perceptual-motor performance in postural sway [12], [16], [18] and walking tasks [8], [18]. Some studies have reported that textured material effects seem to be dependent on the combined influences of two or more sample- or task-related independent variables - such as age, vision or task stability - suggesting that the relationship between added textured and task performance is somewhat complex.

Additionally, despite early work revealing that structural characteristics of texture (i.e. nodule density) can affect the scale of postural sway in upright balance [1], characteristics of textured materials (i.e. internodule distance, width, height, shape, uniformity, hardness, compound type) and related equipment (shoes, insoles, orthotics, socks etc) have varied widely across studies [1], [7], and in some cases have gone unreported [13], [23], [24] or uncontrolled [8], [9], [15], [17]. It is possible, therefore, that inconsistencies in outcomes across studies may be due to variations in treatments, age, health status and experimental task designs reported across studies [40], [45], [47], [48].

The primary aim of this paper is to investigate how added texture has influenced perceptual-motor performance in the different populations studied in previous work. Additionally, we sought to evaluate whether variations in methodology and sample characteristics might have influenced reported effects on perceptual-motor performance by process of systematic review and meta-analysis.

To the best of our knowledge this is the first meta-analysis on the effects of textured materials on measures of perceptual-motor performance. Previous reviews related to somatosensoty facilitation have typically had alternative foci, such as evaluating overall footwear design features [40], [45], [47], [48] (e.g., insole hardness, tread characteristics etc.) or the use tapes or joint support for injury prevention [49], [50]. Given that reviews that have discussed textured materials provide support for their utility, it was anticipated we would confirm that perceptual-motor performance is improved by added texture. Results, although context dependent, were largely favourable and open a number of future research pathways.

## Methods

### Search Strategy

We searched MEDLINE and EMBASE for published primary sources. Ten keywords related to textured material were pooled (via Boolean operation “OR”) and combined (via Boolean operation “AND”) with twenty similarly pooled keywords related to perceptual-motor system functionality. Results were limited to human participants and each database was searched from their earliest available record up to August 2012. We also undertook a related articles search on Google Scholar and carefully scrutinized cited articles and reference lists of all included studies. Articles were restricted to those written in English.

### Inclusion Criteria

No restrictions were made on study design, comparison group or participant sample. For inclusion in the review, studies were required to have involved human participants experiencing treatments with a localized textured material intervention (whose nodules or indenting structures could be described) during performance of a perceptual or perceptual-motor task during which observations were recorded. Textured materials that utilized a power source, such as a vibration-inducing mechanism were excluded, as were interventions where the textured material in some way restricted the range of motion in underlying structures. The former studies involved complex technologies with a mechanism that has been described as stochastic resonance, requiring random signal oscillations utilising a power-source [51]. The latter studies were excluded because restricting the range of motion of body joints might have modified the strategic actions of participants. Finally, studies using adhesive tapes, where no measurable nodules or indenting structures were present, were also excluded. Secondary criteria were developed for inclusion in the meta-analysis. In addition to the primary criteria (required for inclusion in review, see Table S1), studies were required to report sample sizes as well as means and standard deviations (or derivatives) of perceptual-motor performance responses whilst interacting with textured materials and under control conditions without added texture (either dependent or independent groups).

### Selection of Studies

Two authors independently selected trials for inclusion. Titles and abstracts of publications obtained with the search methodology were screened and all studies classified as relevant were retrieved. We utilized a standardized form to select studies eligible for inclusion in the review and/or meta-analysis. Disagreement was resolved by consensus amongst the authors.

### Data Extraction and Management

Data were extracted independently by the lead author and a research assistant using a customized form. This was used to extract relevant data on experimental design, sample characteristics, interventions (including detailed characteristics of texture intervention) and controls, movement task characteristics, independent variables and levels, outcome measures and equipment, and comparisons and interaction effects (see Table S1). There was no blinding of information on lead author or journal outlet at this stage.

### Measures of Treatment Effect

For each study, unbiased (Hedges’ *g*) standardized mean differences (SMD) and 95% confidence intervals (CI) were calculated for continuous outcomes [52]. Effect size estimates for texture compared to no-texture conditions in each study were standardized using the control group standard deviation in dependent group designs [53] and the pooled standard deviations in independent group designs [54]. The synthesized (by average) [55] effect size estimates were, in the first instance, used to calculate the unbiased SMD [52], which were then used to calculate the unbiased variance estimates with equations specialized for variations due to dependent [52] and independent [54] study designs. One study involving repeated measures provided data enough to determine rho values on dependent measures [10]. The rho estimates were then averaged together and used for subsequent calculation of all estimates for the unbiased effect size variances [52]. There were a small number of cases where mean and standard deviation data were deemed unsuitable because of difficulty in interpreting the appropriate direction of the effect [13], [19], [20], [22] or were unobtainable [9], [18], in which case they were excluded from consideration for meta-analysis to avoid contamination. Significance for testing the null (no effect) was set at the one-tailed P<0.05 in favour of texture and utilized the Z-test method [56].

### Risk of Bias

For all studies, methodological quality was assessed independently by the lead author and a research assistant using the Cochrane risk-of–bias tool [57]. Each study was graded on the following domains: sequence generation, performance blinding (participants and personal), assessment bias (detection bias) and for incomplete outcome data (attrition bias). For each study, the domains were determined based on the published study report and judged by the assessors as to their risk of bias. They were assigned the label ‘low’ if criteria for a low risk of bias were met, or ‘high’ if criteria for a high risk of bias were met. If there was insufficient detail to determine either a ‘low’ or ‘high’ risk of bias, then risk of bias was deemed ‘unclear’ for that domain. Disagreements between independent assessors (two authors) regarding risk of bias for domains were resolved by consensus.

### Subgroup Analysis

Differences in study samples and task constraints were anticipated as potential sources of heterogeneity and considered for subgroup analysis. Points of clear differentiation in task constraints were discerned in study details through process of systematic review (Table S1). Different populations were identified by applying MEDLINE age group criteria (all child: 0–18 years (yrs), adult: 19–44 yrs, middle aged: 45–64 yrs, and elderly: 65 yrs and over).

## Results

**Figure 1:**
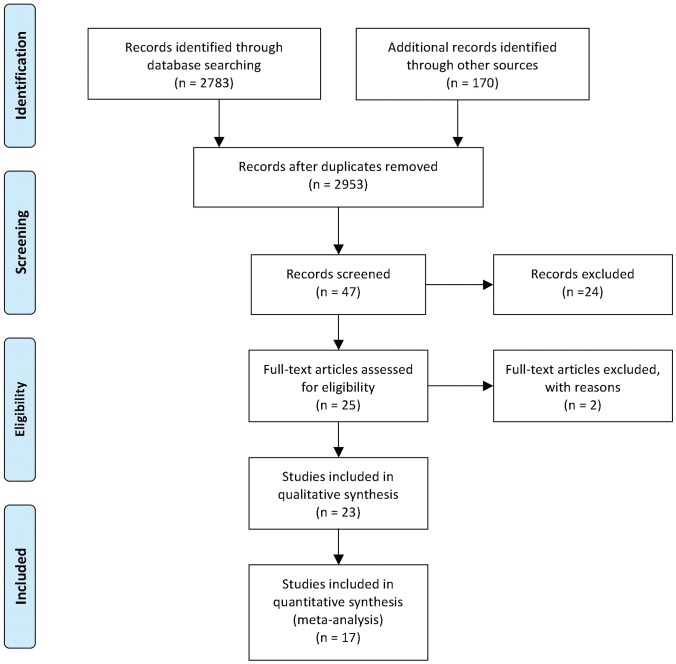
Summary of the search strategy and selection process based on included and excluded studies.

Figure 1 summarises the search strategy and selection process based on included and excluded studies.

### Included Studies

Individual characteristics of included studies are summarised in Table S1. There were 23 eligible studies [1]–[19], [21]–[24], comprising 21 published peer reviewed research articles and two published conference proceedings [9], [13]. Of the excluded studies, notably one was a special issue [20] reporting additional findings on a previously published sample [22] and, therefore, not included in our analysis. Details of sample characteristics of included studies are summarized in Table 1 and show that two distinct age groupings were identified (young from 18 [23] to 51.1 [18] yrs and, elderly, from 64.7 [10] to 79.4 yrs [8]) which could be further characterized by the presence of ailment (with or without). Hence four distinct groups were identified within [2], [3], [5], [6], [12] and between studies: young healthy [1]–[5], [14], [16], [17], [19], [21]–[24], young with ailment [13], [14], [15], elderly healthy [2], [3], [5]–[8], [12], and elderly with ailment [9]–[11].

**Table 1:** Sample Characteristics.

Ailment	Age	N	M	F	Age	SD	Max	Min	Note
**No**	**Young**	330	166	164	27.1	4.6	51.1 [18]	18 [23]	
	**Elderly**	247	97	150	71.7	5.6	79.4 [8]	64.7 [10]	
**Yes**	**Young**	80	26	54	37.4	6.6	49 [13]	21.5 [15]	MS [13], [14], CAI [15]
	**Elderly**	86	42	44	71.1	6.2	79 [9]	65.4 [10]	PD [10], PI [11], FH [9]

**CAI = **Chronic ankle instability; **F = **female; **FH** = falls history; **M = **male; **MS = **Multiple Sclerosis; **N = **sample size; **PD = **Parkinson’s disease; **PI** = plantar insensitivity; **SD = **standard deviation.

The MEDLINE age grouping criteria was departed from in a number of respects. Firstly the adult and middle-aged divisions were grouped to form a ‘young’ group. This was done because only two studies [13], [18], involved participant samples whose mean age fell within the middle-aged group classification according to MEDLINE age grouping criteria. Secondly, a 13.6 yr gap separated the young and elderly age groups and was considered large enough to distinguish between the age groupings, despite the fact that, technically, the by average youngest elderly sample [10] of 64.7 yrs fell inside the cut off for middle-aged classification criteria. It was reasonable therefore to summarize this sample as part of the elderly group. Finally only two studies reported samples of athletes [23], [24], which involved a total of 29 female participants with a mean age of 21 yrs. These athlete participants, due to their small number, were incorporated into the young and healthy subgroup summarised in Table 1.

Five studies [2], [3], [5], [6], [12] were designed with age (young versus (vs.) elderly) as an independent variable, whilst two studies utilized the presence or absence of an ailment as an independent variable [10], [14]. The average sample size was 23 with the largest study based on 80 participants [10]. Twenty-two studies (n = 11 repeated measures, and n = 11 mixed model designs) incorporated at least one textured-intervention group and a no-texture control group. Only one study was observational in that it measured participant’s behaviours with no control group or condition for comparison [13]. Only five studies used a pre-post test protocol where texture conditions were measured on both occasions [2], [8], [11], [13], [18]. One of these studies involved a five-minute between measures period [2]. The time between tests in the remaining studies ranged from 14 to 84 days [8], [11], [13], [18]. One study was unique in that it used a pre-post test design but the first test was always the control condition (no texture). After five minutes a follow-up test with added texture was undertaken and then two additional follow ups under control conditions were then carried out with 5 minutes of either walking or standing between tests [3].

### Risk of Bias

**Figure 2:**
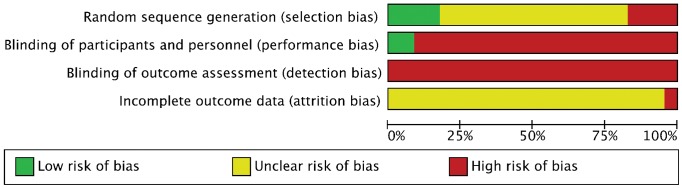
Risk of bias summary.

There was a risk of bias across all 23 studies as summarised in Figure 2. There was a high risk of selection bias in four studies in administering the treatment: three failed to address whether a randomization procedure was in place [4], [21], [23], and one reported that no randomization occurred [19]. Four studies had a low risk of bias, reporting acceptable methods of randomization [7], [8], [10], [16], whilst in the remaining 15 studies the methods of randomization were not reported and received an uncertain risk of bias rating. In both blinding categories (performance and detection), there were high risks of bias across all studies with the exception of two studies that included a sham insole condition [15], [18] and were subsequently rated a low risk of performance bias.

A single study was rated at a high risk of reporting bias because it failed to report outcomes on dependent variables described [19]. The remaining studies were rated at an unclear risk of attrition bias for not reporting methods for handling participant drop out and/or not providing methods allowing judgment of whether the pre-planned dependent variables were reported.

### Details of Intervention

Twenty-two studies applied texture to some part of the plantar surface of the foot [1]–[11], [13]–[19], [21]–[24], and one study was unique in adding texture, in a uniform arrangement, to the right index finger tip [12]. Sixteen of the studies that applied texture to the feet did so to the entire plantar surface [1]–[5], [7], [8], [14]–[19], [21], [23], [24], three of which were in a non-uniform pattern [2], [3], [8]. Five studies applied texture to select aspects of the plantar surface (all with a uniform structure). Three of these studies applied it only to the perimeter of the plantar surface [6], [10], [11], one study involved a condition where texture was applied to the middle third of the plantar surface [19] and one applied it longitudinally to the medial third of the plantar surface [22]. Two studies did not report enough information to determine the extent and consistency of the textured material applied to the foot surface [9], [13].

The majority of studies used a textured insole in shoes worn by participants (n = 14 [5], [8]–[11], [13]–[15], [17], [18], [21]–[24]). Of the remaining nine, five studies applied the texture to a standing surface [1], [4], [7], [12], [16], two studies used a textured sandal [2], [3], one study glued gravel to the inside of a sock [19], and another study used adhesive tape to fix a flexible tube to the border of the plantar surfaces [6]. The texture in all non-insole studies made direct contact with the participants’ skin surface (i.e. no intervening material was included between the texture and skin surface although, the studies involving textured sandals did not explicitly report this [2], [3]).

Of the insole studies, 12 inserted the insole into a pair of shoes [8]–[11], [13]–[15], [17], [18], [22]–[24]. Of these shod studies, five controlled for shoe type [10], [11], [14], [18], [22], four used the participants’ personal shoes [8], [9], [15], [17], and three did not report whether the shoes were controlled or personal [13], [23], [24]. Five reported using socks [15], [17], [18], [23], [24], four of which reportedly controlled the type of sock worn between the in-shoe textured insole and skin [15], [17], [18], [23]. Three studies did not report use of socks but, very likely, involved the participants’ personal socks, given that the textured in-shoe insoles were worn by participants for a time period in the range of 14 to 84 days [8], [11], [13]. There were two studies involving shoes that did not report whether or not socks were used [9], [10] and only two studies involving shoes reported not to have used socks (i.e. textured insoles were in direct contact with the skin) [14], [22]. The two studies involving insoles that did not use shoes had unique intervention characteristics. One study observed participants standing directly onto textured insoles positioned on the surface of a force platform [5] (i.e. as a textured insole surface), whilst one study used an adhesive wrapping over the insoles and foot, recording participant performance whilst walking with direct contact with the insoles [21].

Of the studies that applied texture to a surface, four studies applied texture over a force platform that participants stood on whilst performing an upright balance task [1], [4], [7], [16], and one study applied texture to a surface positioned at hip height that participants touched with their right index finger during an upright balance task [12].

Seventeen studies used a single texture type [2]–[6], [8]–[12], [14], [15], [17], [21]–[24], five examined two different types [7], [13], [16], [18], [19], and a single study compared three types [1]. A range of materials (rubber/foam/plastic [2]–[7], [10], [12], [13], [16]–[18], [21], [23], [24], sandpaper [14], gravel [19], leather [8] and metal [1]) and shapes (points/spikes [2]–[4], [7], [15], [16], [18], rounding [1], [5], [17], [22]–[24], semicircles [7], [16], [18], [21], grooves [8], [12] or ridges [6], [10], [11]) were described. The majority of studies [1]–[7], [10]–[12], [14]–[19], [21]–[24] also reported measures of the structural characteristics of texture applied (including internodule distance/density [1]–[4], [7], [12], [15], [21]–[24], height [1]–[6], [10], [11], [17], [23], [24] and/or width/diameter [2]–[4], [12], [14], [19], [22]), as summarized in Table 2, in descending order of nodule magnitude.

**Table 2:** Texture Dimensions and Characteristics.

Study	Density (n/cm^2^)	Dist. (mm)	Height (mm)	Width (mm)	Shape or material
*(Hatton et al., 2011) [7] a.	nr	2.5	nr	nr	point
*(Preszner-Domjan et al., 2012) [4]	5	nr	7	2	spike
*(Waddington et al., 2000) [23]	4	nr	7	nr	round
*(Waddington et al., 2003) [24]	4	nr	7	nr	round
*(McKeon et al., 2012) [15]	4	nr	nr	nr	round
*(Hatton et al., 2011) [7] b.	nr	5	nr	nr	semicircle
*(Palluel et al., 2008) [2]	3	nr	5	3	spike
*(Palluel et al., 2009) [3]	3	nr	5	3	spike
(Nurse et al., 2005) [25]	nr	8	nr	nr	semicircle
*(Watanabe & Okubo 1981) [1] a.	nr	10	1	nr	round
(Ritchie et al., 2011) [22]	nr	12	nr	4	round
*(Watanabe & Okubo 1981) [1] b.	nr	15	1	nr	round
*(Watanabe & Okubo 1981) [1] c.	nr	20	1	nr	round
*(Corbin et al., 2007) [17]	nr	nr	2.5	5.5	round
*(Qui et al., 2012) [5]	nr	nr	3.1	5	round
(Chen et al., 1995) [19] a.	nr	nr	nr	5.5	gravel
(Chen et al., 1995) [19] b.	nr	nr	nr	2.5	gravel
*(Maki et al., 1999) [6]	na	na	3	3	ridge
*(Jenkins et al., 2009) [10]	na	na	2	nr	ridge
*(Perry et al., 2008) [11]	na	na	2	nr	ridge
*(Tremblay et al., 2004) [12]	na	2.7	na	2.5	groove
*(Kelleher et al., 2010) [14]	na	na	na	0.2	sandpaper
*(Hatton et al., 2009) [16] a.	nr	nr	nr	nr	point
*(Hatton et al., 2009) [16] b.	nr	nr	nr	nr	circle
(Wilson et al., 2008) [18] a.	nr	nr	nr	nr	point
(Wilson et al., 2008) [18] b.	nr	nr	nr	nr	circle
*(Hartmann et al., 2010) [8]	nr	nr	nr	nr	leather
(Dixon et al., 2012) [13] a.	nr	nr	nr	nr	nr
(Dixon et al., 2012) [13] b.	nr	nr	nr	nr	nr
(Hatton et al., 2012) [9]	nr	nr	nr	nr	nr
Average	3.83	9.40	3.58	3.29	

* = studies selected for meta-analysis, **Dist.** = Distance **n/cm^2^** = nodules per centimetre squared.

Of the seven studies [1], [5], [7], [13], [16], [18], [19] that used texture as an independent variable (having at least two texture modifications), significant differences in performance measures were reported in a single study [13] (two additional studies described differences [1], [19]). It should be emphasized that only Watanabe and Okubo [1] and Chen et al. [19] altered packing density and the remaining studies altered the pattern of the surface texture. Of note also is that Qiu et al. [5] manipulated the hardness (soft vs. hard) of a textured insole and found significant interactions with performance effects favouring the soft insole condition, under more unstable foam surface standing conditions for elderly participants however, favoured hard insoles for young participants (eyes open and eyes closed).

### Summary Effects of Texture

**Figure 3:**
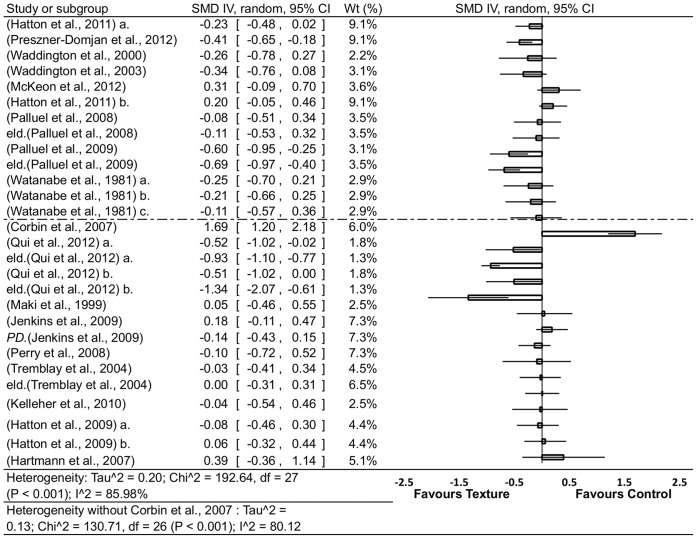
Forrest plot summary of the effect of textured material on perceptual-motor performance. **CI** = confidence interval; **eld.** = elderly group; **PD** = Parkinson’s disease; **IV** = inverse variance; **random** = randomized; **SMD** = standardized mean difference; **Wt** = weight. Notes: within groups vs baseline and between [8], [11] groups vs baseline. The first dashed line from the top includes groups where the internodue distance was known and are in order starting with the smallest to largest internodule disatance. The remaining studies do not report details regarding internodule distance and are in order of how much information was aviliable regarding the texture material characteristics.

The Forrest plot summarizing the effects of textures clearly suggests a trend toward improved perceptual-motor performance and shows a range of strong [5], moderate to strong [3], [5], small to moderate [1], [4], [7], [15], [23], [24], absent [1], [2], [6], [7], [10]–[12], [14], [16] and adverse [8], [15], [17] effects (Figure 3). However, analysis revealed significant heterogeneity across the effect sizes (Figure 3) and identified a very large outlier [17]. After removal, heterogeneity was still confirmed, suggesting that there were potentially significant influences by subgroups, either at population, task or experimental control level. Finally, except in the case of Watanabe and Okubo [1], the data suggested no clear trend when ordered by descending order of nodule density.

### Details of Performance Outcomes

Prior to undertaking the meta-analysis, it was anticipated that task factors might play a role in the variability across studies. Hence, the following sections describe the interventions at a level of detail that uncovers task related features inherent to the strategies for observing perceptual-motor behaviour employed across studies.

Twelve studies recorded centre of pressure excursions [1]–[7], [12], [15]–[18] with an average extent time of 28.4 s, (minimum 10 s [15], [17], maximum 60 s [12]). All studies of this type used a force platform and reported a variety of measures: area covered (mm^2^) [1]–[3], [5], [17], excursion length (mm) [1], [5], excursion velocity (mm/s) [2], [3], [7], [17], anterior-posterior (mm) [1], [5], [7], [12], [16], [18], root mean square [2], [3], Hz [2], time to boundary (s) [15] and/or medial-lateral (mm [1], [5], [7], [12], [16], [18], root mean square [2], [3], Hz [2], time to boundary (s) [15]) sway parameters. Three studies measured surface electromyography of lower limb muscles [6], [7], [16]. One study also measured ground reaction forces, frequency and distance of stepping actions induced by perturbations during an upright balance task [6]. Furthermore, one study took measures of finger-tip force [12], and one took measures of tibial nerve discharge [1]. In studies that utilized the standing balance paradigm, five independent variables were identified including: vision (eyes open [1], [4]–[7], [12], [15]–[18], eyes closed [1]–[7], [12], [15], [17], [18]), follow up (pre vs. post conditions [2], [3], [18]), and surface (all involved a hard surface condition but only three involved a foam surface condition [4], [5], [12]). One study used perturbation methods including intermittent and continuous destabilization of the support surface and a dual task [6], and one study manipulated breathing [1] (normal vs. held breath).

Ten studies observed effects of added texture during walking [8]–[11], [13], [14], [18], [19], [21], [22], with one study including a sub-condition that involved running gait [19], which was also unique in using a treadmill. The majority of studies sought participants to walk at a self-preferred pace, with only two studies requiring a set pace be met (5.4 [21], 6 [19] and 13 km/hr [19]). The average reported distance that participants were required to walk was 12.0 m (minimum distance = 6.1 m, maximum = 24 m). Locomotion was typically observed on level and hard surfaces with the exception of two studies [8], [11] that modified the surface slope [11] and compliance [8]. A variety of gait parameters were reported including spatiotemporal (walking velocity (m/s) [8]–[10], [13], [14], cadence (steps/minute) [8], [9], [13], [14], stride/step length (m) [8]–[10], [13], distance to base of support (m) [9], support duration (s) [8]–[10] and step length variability (mm) [10]), kinematic (midfoot-tibial angle [21], [22] and hip/knee/ankle absolute angles [14], [21]), kinetic (ground reaction force [14], [21] and knee/ankle joint torque [21] or foot sole pressure distribution [19]) characteristics. Four studies [9], [10], [13], [18] utilized an instrumented walkway to record spatiotemporal features of gait, two used a multiple camera array [14], [22] and one used triaxial accelerometers [8]. All four studies to report lower limb segment kinematics utilized a multi-camera array [11], [14], [21], [22], and kinetic data were collected using camera and force plate technology [14], [21]. The single study to report pressure distribution patterns utilized a pressure-measuring insole [19]. Finally, lower limb electromyography data were collected across four studies [10], [14], [21], [22].

Four task-related independent variables were identified in studies requiring bipedal locomotion task performance; time (pre, post) [8], [11], [13], [18], surface (hard [8]–[10], [13], [14], [18], [19], [21], [22], compliant [8], slope/uneven [11]), cognitive load (neutral [8]–[11], [13], [14], [18], [19], [21], [22], dual task [8]) and movement patterning (walking [8]–[11], [13], [14], [18], [19], [21], [22], running [19]).

Two studies were unique in utilizing a psychophysical paradigm to assess texture on perception (absolute judgment) of five different joint positions of ankle inversion [23], [24].

### Stability in Upright Balance Tasks

Twelve studies [1]–[7], [12], [15]–[18] focused on postural stability in upright balance tasks. Eight of these studies [1]–[7], [17] reported significant reductions interpreted as improvements in postural sway parameters with added texture and two studies reported changes consistent with an adverse effect for texture [15], [17]. Main effects for texture were reported in six studies [1]–[3], [5], [6], [15], and interactions were reported across seven studies for vision [4], [7], [15], [17], age [2], [3], [5], time [2], [3], surface hardness [4], [5], number of supporting legs [17] and resting task requirements [3].

### Performance in Bipedal Locomotion Tasks

Ten studies [8]–[11], [13], [14], [18], [19], [21], [22] reported outcomes on measures recorded during performance of bipedal locomotion tasks. Six of these studies [9]–[11], [14], [21], [22] reported significant changes in performance measures (vs. control) that were interpreted as beneficial. Main effects were reported in five studies, and interactions were reported in one study [10] for trial, gait cycle phase and ailment.

#### Summary effects of task

**Figure 4:**
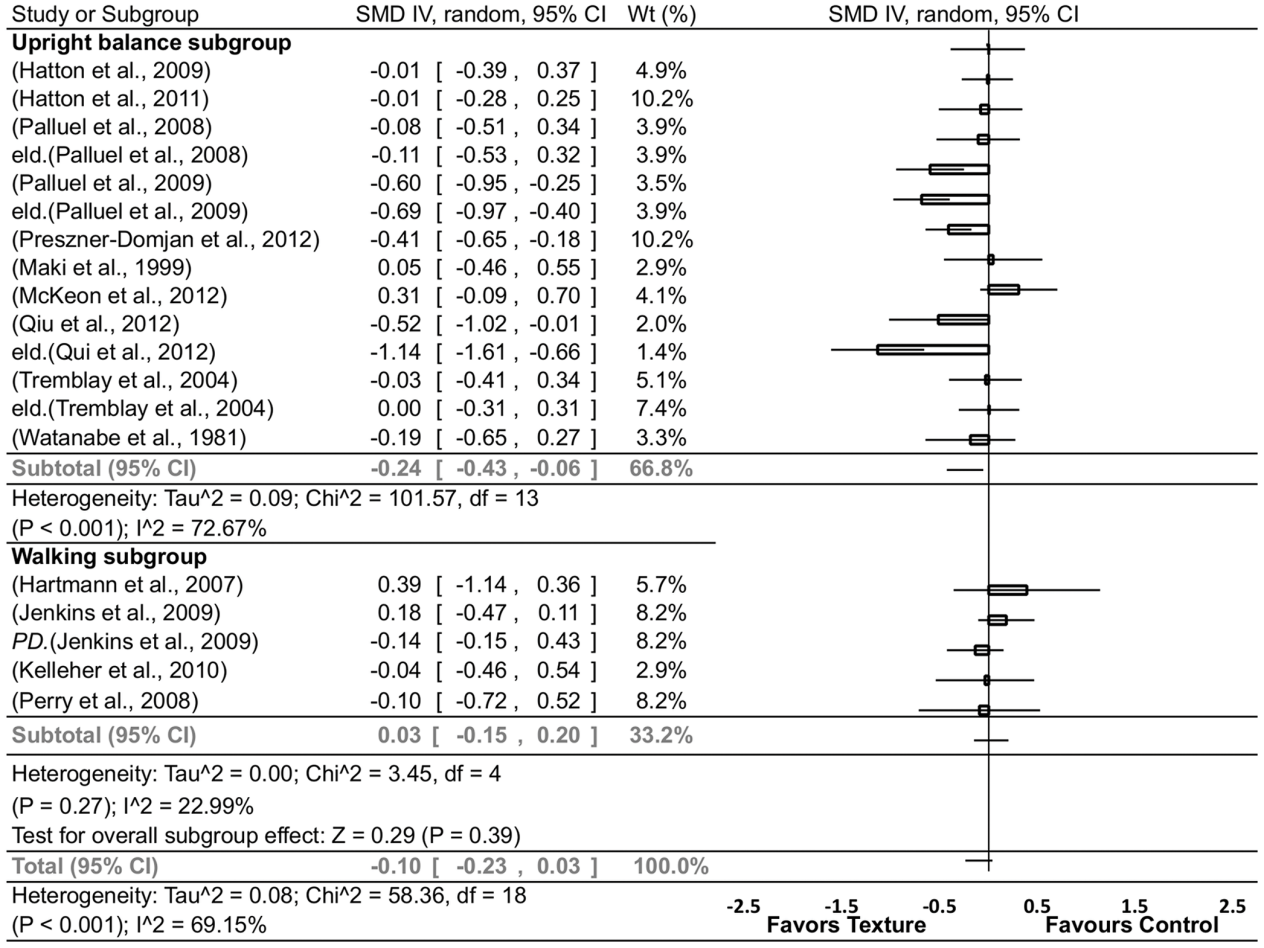
Forrest plot summary of textured material effects on perceptual-motor performance in upright balance or walking tasks. CI = confidence interval; eld. = elderly group; PD = Parkinson’s disease; IV = inverse variance; random = randomized; SMD = standardized mean difference; Wt = weight. Note: within groups vs baseline and between [8], [11] groups vs baseline.

Across the included studies, two clearly distinguishable perceptual-motor task vehicles for evaluating the effects of texture on motor performance were upright balance and walking. We analysed the effect size estimates of each study involving either an upright balance or walking task and found significant heterogeneity in the pooled effect sizes of the upright balance conditions (Figure 4). Conversely, significant homogeneity was found in the pooled effect sizes of the walking conditions (Figure 4), which, nonetheless, showed no effects in favour of or against texture. Furthermore, the overall pooled effect sizes also showed significant heterogeneity.

**Figure 5:**
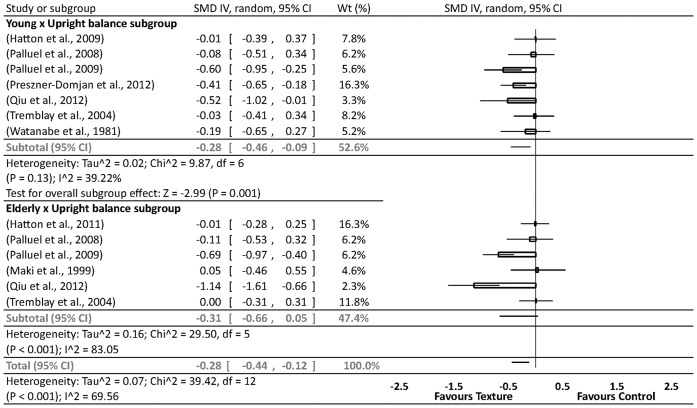
Forrest plot summary of textured material effects on perceptual-motor performance in upright balance tasks – age grouped. CI = confidence interval; IV = inverse variance; random = randomized; SMD = standardized mean difference; Wt = weight. Note: within groups vs baseline.

The walking group sample characteristics may have underpinned the homogeneity in summary effects. Of the four, three studies [8], [10], [11] involved elderly participants (n = 176) with an average age of 70.6 yrs (range = 79.4–65.1 yrs), one of which involved a subgroup with Parkinson’s disease [10]. Another study involved participants who had been diagnosed with plantar insensitivity [11]. The fourth study was of a group of 14 middle-aged adults (average 41.8 yrs) diagnosed with both multiple sclerosis (MS) and plantar insensitivity [14]. In contrast, the upright balance effect size summary included both young [1]–[5], [12], [15], [16] (n = 181, 25.1 yrs) and elderly [2], [3], [5], [6], [12] (n = 145, 70.5 yrs) participant samples and also had one study that involved a group with chronic ankle instability [15]. To determine whether sample characteristics were related to heterogeneity, we subsequently undertook a subgroup analysis of age utilizing only the upright balance data from healthy participants (Figure 5).

#### Summary effects of age, vision and stability in upright balance tasks

Homogeneity was found in the young subgroup in addition to significant small to moderate subgroup effects in favour of texture for improved perceptual-motor performance (SDM = 0.28, Figure 5). In contrast, however, the elderly subgroup analysis showed significant heterogeneity suggesting that task or experimental design-related factors were influencing levels of variability across effect sizes (Figure 5).

**Figure 6:**
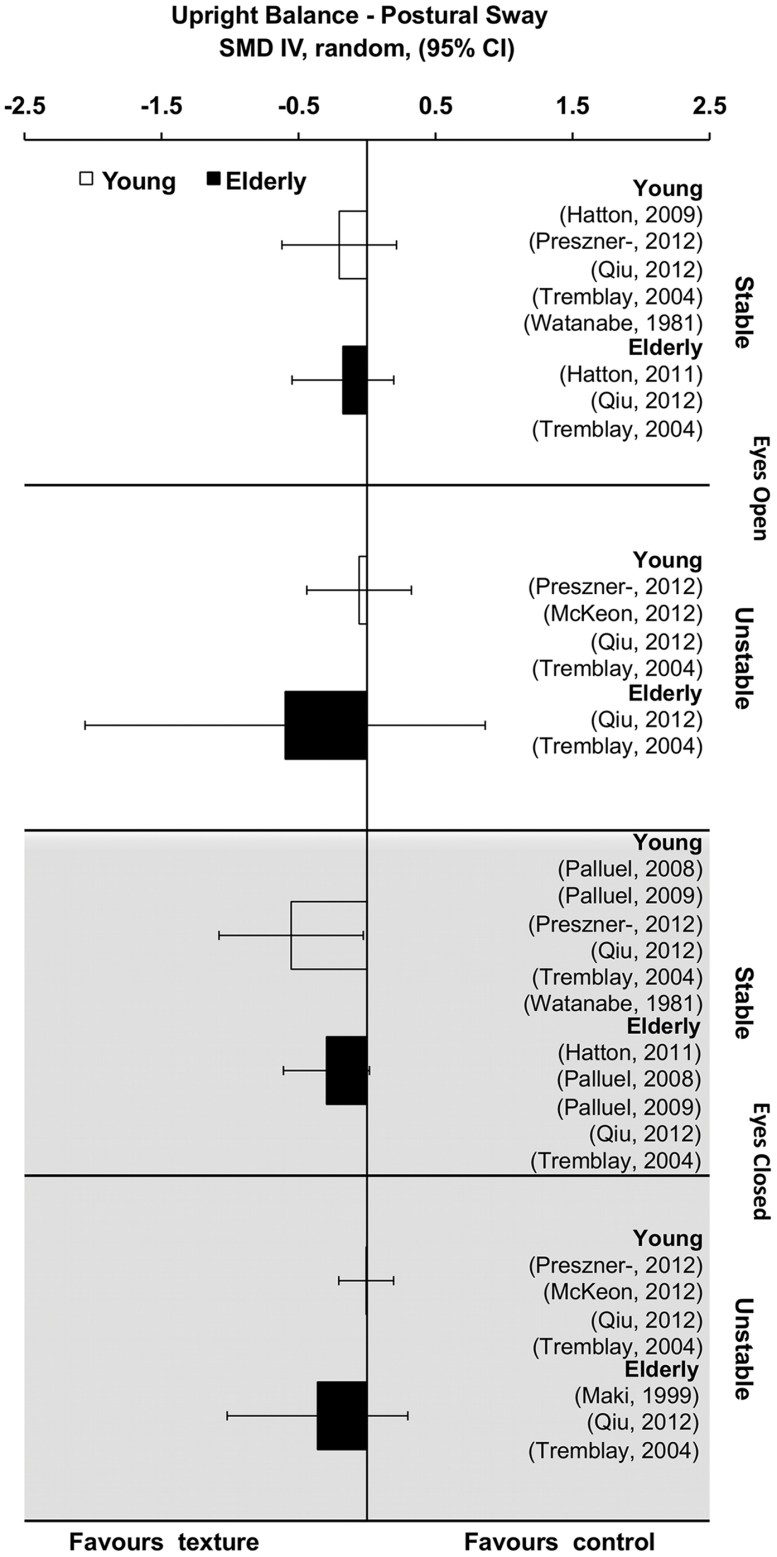
Pooled summary effect sizes in upright balance tasks grouped by ‘common’ experimental design constraints. CI = confidence interval; IV = inverse variance; random = randomized; SMD = standardized mean difference.

To highlight variations in task design variables we next calculated summary effect sizes for different levels of vision (eyes open and eyes closed) and stability (stable and unstable) for each subpopulation (young and old), the results of which are sumarised in Figure 6. Whilst, it was not feasable because of the small number of studies comprising subgroups, this qualitative analysis may provide a platform for organising studies as more findings are published. For young participants, two conditions, eyes closed by stable and eyes closed by unstable, revealed pooled effect size estimates that appear meaningful (SMD = 0.20 and 0.55 repectively). Furthermore, for elderly participants, on the other hand, three conditions, eyes open by unstable, eyes closed by stable and eyes closed by unstable, displayed pooled effect size estimates that suggest meaningful values (SMD = 0.60, 0.30 and 0.36 respectively).

### Follow-Up

Six studies utilized a pre-post test design [2], [3], [8], [11], [13], [18]. Three studies [2], [3], [11] reported significant improvements in performance measures with added texture (vs. control). Perry et al. [11] required an experimental group of participants to wear textured inserts for 12 weeks. However, they showed that in the experimental group, performance measures, whilst significantly better in the pre-test, were not significantly different (vs. control) in the post-test. Intriguingly, this was because performance under the no-texture condition increased to levels similar to the with-texture condition. Also in this study, during the 12-week period, 12 participants reported falling events, nine of which were in the control group. Palluel et al. [2] and Palluel and Nougier [3] observed young and elderly participants under eyes closed and double limb support conditions, with both reporting an overall significant reduction in centre of pressure sway area (vs. control) regardless of age. Palluel et al. [2] reported a significant interaction for age and resting task. Similar reductions in centre of pressure sway area were reported for older and younger participants after five minutes of *standing stationary* and *walking* between postural sway tests, respectively. Palluel and Nougier [3] replicated these findings reporting that standing between sessions was more beneficial for elderly participants and walking more beneficial for young participants. However, this study differed in that during rest periods - involving standing still or walking - participants wore the textured material.

## Discussion

### Using Texture for Improved Perceptual-motor Performance

#### Material characteristics

The purpose of introducing texture is to enhance the sensory input from regions of indentation. The proposed mechanism for the effects involves an increase in the rate of discharge from stimulated groups of cutaneous receptors. Indentation or stretch at sufficient intensity provides information about characteristics of the material, such as roughness, spatial resolution and orientation. However, it needs to be resolved whether greater quantities or amplitudes (depth) of indentations caused a greater number of receptors to fire and at higher rates [16], [41], the data clearly suggests the relationship is not so simple. Additionally, because characteristics such as shape, contouring or hardness might influence the degree of deformation of skin receptors, it is reasonable to expect that these factors too would influence receptor stimulation to some degree [5], [7]. The data presented in this review showed that whilst texture enhanced perceptual-motor functionality, effects were dependent on contextual factors such as individual, environmental and/or task constraints and were strong enough to distort any systematic effects of packing density previously shown by Watanabe and Okubo [1].

#### Upright balance and age

Regulation of the centre of mass enables people to maintain upright posture and is believed to be a key factor for falls risk in elderly participants [58]. Because ageing negatively effects structural (e.g. receptor morphology) and functional (e.g. nerve condition velocity) components of the somatosensory system, texture is introduced on the basis that it can stimulate peripheral receptors that are otherwise not being stimulated [5]. Indeed, older adults have been observed to have increased muscle activity in leg muscles, suggesting a reliance on conscious strategies for postural stability [7], [18], a conclusion supported by observations of more deterministic patterns of postural sway in older adults by other research groups [59], [60].

It is clear that further research in elderly populations is required, for instance studies by Qiu et al. [5], Tremblay et al. [12] and Maki et al. [6] comprised the only efforts to evaluate effects of texture on upright balance under unstable postural conditions. Certainly the moderate to large summary effect sizes in favour of texture (0.52) shown in the study by Qiu et al. [5] warrant further evaluation. This is especially the case given the design differences between the studies of Qiu et al. [5] (who used foam to induce instability with texture added at foot sole) and Maki et al. [6] (where perturbations were induced by moving the platform and including a dual task), and Tremblay et al. [12] (who used foam to induce instability, but the finger tip was the point of texture contact as opposed to the foot sole in Qiu et al. [5]).

Nonetheless, the data unequivocally show that during upright balance tasks, the largest and most reliable effects in favour of added textured material were observed in young, healthy individuals (0.28). Furthermore, these favourable effects were strongest under conditions where reliance on somatosenory system information was exacerbated by removal of visual information (0.55). Therefore, textured material effects, here, support the notion that interaction with texture improves body awareness and specifically the spatial representation of the pressure distribution at the foot sole [2], [3]. It may be for instance that an external focus of attention was implicitly supported, a combination previously associated with improved perceptual performance [61].

These findings are also in line with data reported by Waddington and Adams [23], [24]. In two studies, young athletes wearing socks and shoes with textured inserts were able to discriminate between five different joint positions with success equivalent to barefoot conditions. Their sensory discrimination performance was significantly better than when wearing shoes with no added insoles and socks.

However, it should be noted that there exist potentially separate mechanisms to explain why young participants display enhanced joint position discrimination, which might be considered a mainly sensory detection problem, as opposed to a coupled, perceptual-motor problem, as in the case of postural regulation. Furthermore, the mechanisms for these reported somatosensory facilitation effects remain unknown [2], [62] and were a question beyond the scope of the current study.

#### Walking

Alteration to sensory inputs at the foot sole have been clearly shown in movement kinetics and kinematics across studies in this review [10], [11], [13], [14], [19], [21], [22] with the exception of data from Wilson et al. [18]. The collective weight of these findings support the notion that sensory feedback from cutaneous receptors in the foot sole is involved in determining movement strategies during walking [21] or during recovery actions such as stepping to recover balance [6].

Taken in conjunction with the results of the meta-analysis, however, some doubts emerge on the efficacy of added texture in healthy elderly participants with only one study demonstrating any meaningful effect [8], which was not in favour of an intervention. Furthermore, there remains doubt about which direction changes in outcome measures, such as walking speed, reflect a functional improvement. For instance, in healthy young participants, typically increases in gait variables such as walking velocity, cadence and step length are interpreted to the functional direction of effect. Contrary to these observations, however, a number of studies reported reductions in gait variables [9], [14], but were nonetheless interpreted as functional changes on the basis that the participants were either elderly [9] or diagnosed with MS [14] and hence, reflected and increased level of caution.

### Long Term Usage

Textured materials appear to be utilized continuously by participants in that, after their removal, performance deteriorates back to baseline levels [3]. Intriguingly, in the study by Perry et al. [11] measures taken during walking over a series of uneven platforms showed that, after 12 weeks of resistance training, performance began to converge between the group that wore textured insoles for the 12 week period and the group that did not. Perceptual-motor stability has been observed previously in muscles that underwent four weeks of resistance training. Improvements in measures of coordination stability were observed (compared to muscles or participants that did not undergo training) [63]. The suggestion then, is that stability in perceptual-motor performance may be facilitated through textured material, and/or resistance training programs.

### Advantages and Disadvantages for Using Textured Materials

The principal advantage of using textured materials appears to involve facilitating the tighter regulation and control of spatial and temporal characteristics of the centre of mass over an individual’s base of support. Improvements in the ability to detect information changes, such as changes in balance [5], [7] or the positioning of a limb [24], might help prevent falls [8], [11] and injuries [23], [24] or facilitate the perception of useful information, supporting adaptive regulation of movement [6], [10].

There appear to be no disadvantages regarding use of textured materials that cannot be overcome and preclude their utilization. There are, however, two concerns raised in the literature that warrant consideration in future research, product development or application: comfort [2], [5], [11], [15], [24] and time course of sensory re-calibration [2], [3], [10], [11], [15].

Comfort is an important research, design and clinical issue. It is recommended that future work be guided by the nature of environmental and task-related constraints that will determine how textured materials will be interacted with. To exemplify, the design needs of a textured surface for an athlete who might perform repetitive and forceful contacts with a textured surface will be different to those of an elderly individual undertaking daily activities or of a patient with peripheral neuropathy who may have ulcers and wounds on the foot surface [5], [11]. Some studies have reported participant discomfort with the use of textured materials [2], [5], whilst, one 12 week study reported no occurrences of discomfort associated with textured material use. To ameliorate discomfort, design strategies can include utilizing soft insole material [5], strategically placing texture [6], [11] or utilizing customized devices such as orthotic insoles [17].

With regards to sensory re-calibration, a number of experimental and clinically relevant recommendations can be provided. There is some evidence that a short period of exposure (through sustained contact or activity) is important in order that improvements in performance with added texture might take effect [five minutes of contact or four trials can be recommended, 2,10]. In addition, when participants remove insoles, there is evidence that performance deteriorates for a short period of time [3]. These phenomena are believed to reflect a short term process of sensory re-calibration [3], and hence it is recommended that in future research, and in applied settings, that users are allowed a period of at least five minutes pre- and post-usage in order to benefit from the textured material, and to re-adapt in cases of it’s withdrawal. It should also be noted that concerns for potential long term habituation (i.e. an adjustment in the resting sensory detection threshold) with added texture appear to be unfounded, with long term studies suggesting no such effects [11].

Furthermore, there appear to be interactions with disease factors that are currently not fully understood [10], [14], [15]. McKeon et al. [15] found that performance in individuals with chronic ankle instability reduced performance with added texture in a single limb stance task (vs. control). Although, in this study no time was provided for participants to become accustomed to the texture, the findings suggest that, in the presence of chronic ankle instability, performance is reduced [15]. Hence, future research is needed to determine whether this is an acute or long term effect in such individuals.

### Limitations and Future Study

This study undertook a comprehensive search of relevant databases and meticulously assessed supplementary materials. We acknowledge however, that the gray literature (material not formally published) may contain other relevant studies subsequently overlooked. Additionally, no study had a registered protocol and hence accurate assessment of reporting bias was constrained. Finally there were a number of outcomes where summary values were extracted from graphs and although minimization involved two independent reviewers undertaking this, with inconsistencies resolved through third party consensus, the data extracted this way reflects an estimation of treatment effect.

The findings of this systematic review suggest a number of fruitful avenues for future research. There is unequivocal evidence that young, healthy individuals improve perceptual-motor performance with added texture. Furthermore the increased stability in perceptual-motor performance for young participants with short term exposure [2], [3], taken together with data showing maintenance of performance over long time scales in elderly participants [11], provides reasonable evidence that texture is robust to habituation and exploited on-line. It is likely that distinct populations, such as developing elite athletes or young children over the age of eight yrs [64], might also respond to added textured material in contexts to enhance somatosensory perception during learning and development [65]. Additionally, added texture is likely to be valuable in performance of highly constrained movements where visual perception of information is challenging [66], such as in high speed interceptive actions (e.g. kicking, batting, catching etc.), or in the presence of exercise induced fatigue [67]–[69]. Should findings confirm, for example, that with textured insoles, kicking performance improves, then this observation would open the way for examining the functional utility of textured inner garments or texture applied to important objects and surfaces in performance environments. It is also entirely possible that extended time scale benefits of textured material could reveal themselves under transfer and retention tests, or in injury occurrence rates. Furthermore, there may also be an opportunity to combine textured materials with biofeedback techniques currently emerging as a useful method for providing augmented feedback for improving perceptual-motor performance [70], [71]. These various research opportunities are yet to be explored in the extant literature.

Current research efforts have only just begun to explore the role of added texture in populations with perceptual-motor deficits, and indeed there is clear scope for future research in this area. For example, it needs to be understood whether, in clinical groups (e.g. diabetic peripheral neuropathy, Parkinson’s disease or MS), textured materials might provide potentially stronger benefits than in healthy populations. Whilst there is clear supportive evidence across a number of studies, further research with larger sample sizes and under conditions that require reliance on cutaneous inputs is required. Furthermore, the risk-of-bias assessment revealed that there was bias for blinding across studies. Whilst it is acknowledged that it is often difficult to allocate resources necessary to blind assessors or include sham conditions to control for potential placebo effects, future studies should begin to address this issue to improve the quality of work in this area. As a final point, the review uncovered a void of research into the utility of texture in other settings such as work environments, military or hospitals. Adding texture to surfaces or tools may well facilitate improved performance behaviours in these contexts.

### Conclusion

Utilizing methods derived from systematic review and meta-analysis [72] (see ChecklistS1), this study provided clear evidence for the role of textured material in improving perceptual-motor functionality in young healthy individuals and serves as a strong basis for future research with such individuals. Textured material is also likely to be robust to habituation effects and research to evaluate whether it may improve learning will be an important research step. Also discussed is the likelihood that texture materials should improve performance under constraints where high movement speeds reduce opportunity for visual perception, such as in elite sport contexts, but which as yet requires confirmation. Finally, future research with larger sample sizes and more effective experimental control is also clearly required in elderly and clinical populations due to ongoing variability across effect size estimates.

## Supporting Information

Table S1 **Study Characteristics.**
(DOCX)

Checklist S1 **PRISMA 2009 Checklist.**
(DOC)
