# Supplementary material for: The Role of Textured Material in Supporting Perceptual-Motor Functions
Source: PLoS One. 2013 Apr 2;8(4):e60349. doi: 10.1371/journal.pone.0060349 (PMC3615024; doi:10.1371/journal.pone.0060349)
Supplement: Table S1 — Study Characteristics. (DOCX) [file pone.0060349.s001.docx]

**Table S1. Study Characteristics**

| **Study [type]** | **No. of participants; age (y)^a^** | **Intervention^b^** | **Task Design^c^** | **Outcomes recorded [training period]^d^** | **Summary of significant effects^e, f, g^** |
| --- | --- | --- | --- | --- | --- |
| (Chen et al., 1995) [19] [RM] – statistics nr | 10 healthy M; 29.2±5.3 | Plantar surface (direct); textured sock (uniform and nonuniform):  A. control  B. gravel (uniform, 5.5 mm diameter)  C. gravel (middle third of foot, 5.5 mm diameter)  D. gravel (uniform, 2.5 mm diameter)  [shoes provided] | Walking (6 km/hr) and running (13 km/hr); distance nr  [order of treatment not randomized] | Gait (foot pressure distribution); pressure measuring insole:  1. Force (maximum, maximal active area, impulse)  2. Pressure (peak, pressure-time integral) | 1 and 2 increased^f^ and decreased^f^ (specific to localized foot sole regions)  Note: Functionality of changes were not interpreted |
| *(Corbin et al., 2007) [17] [RM] | 33 healthy, 17 F; 27.4±9.1 | Plantar surface (indirect); textured insole (uniform):  A. control insole  B. textured insole (raised plastic nodules: 2.5 mm height)  [participant’s shoes, socks provided] | Upright balance (double); 10 s:  i. EO, EC  ii. double and single support | Postural sway (CoP excursion); force plate:  1. Area (cm^2^)  2. Velocity (cm/s) | No significant findings (vs. A)  [1 and 2 texture x vision interaction^g^ (bilateral condition)] |
| (Dixon et al., 2012) [13] [MM] – conference proceeding, no control group/condition | 46 diagnosed MS, 34 F; 49±7 | Plantar surface ((in)direct nr); textured insole (uniformity nr):  A. textured insole (nr)  B. textured insole (nr)  [shoe type nr] | Walking (self preferred speed); distance nr:  i. pre-post (2 weeks wearing insoles between test) | Gait (lower limb: spatiotemporal); instrumented surface:  1. Cadence (steps/min)  2. Stride-length (m)  3. Walking speed (m/s) | No control group for comparison  Note: 1 increased^f^ in A and B (vs. pre) |
| *(Hartmann et al., 2010) [8] [MM] | 42 healthy: untrained control subgroup (n = 14, 10 F); 76.0±6.0,  trained control subgroup (n = 14, 9 F); 79.4±5.2,  trained texture subgroup (n = 14, 10 F); 76.4±5.9 | Plantar surface ((in)direct nr); textured insole (nonuniform):  A. control insole  B. textured insole (leather, grooves at heel, medial and lateral edges and metatarsals)  [participant’s shoes, socks nr] | Walking (self preferred); 24 m:  i. hard, soft surface  ii. dual task (counting backwards in 3’s)  [pre-post tests for both tasks with 24 resistance training sessions in between in trained subgroups or, with 12 week period in between tests in  untrained subgroup] | Gait (lower limb: spatiotemporal); triaxial accelerometer:  1. Cadence (steps/min)  2. Step duration (s)  3. Step length (m)  4. Walking speed (m/s) | No significant findings (vs. A)  Note: 3 and 4 increased^f^ in trained control and trained texture subgroups vs. untrained control subgroup |
| *(Hatton et al., 2009) [16] [RM] | 24 healthy, 17 F; 27.5±7.5 | Plantar surface; surface (direct); textured surface (uniform):  A. control surface  B. textured surface (pointed nodules)  C. textured surface (semicircular nodules)  [unshod, insoles; 3 mm thick, shore A50] | Upright balance (double, EO); 30 s | Postural sway (CoP excursion); force plate:  1. AP length  2. AP SD  3. ML length  4. ML SD (mm)  EMG; surface (BF, MG, PL, RF, SOL, TA, VL, VM):  5. Amplitude (µV) | No significant findings |
| *(Hatton et al., 2011) [7] [RM] | 50 healthy, 29 F; 75.1±5.0 | Plantar surface (direct); textured surface (uniform):  A. control surface (force plate)  B. textured surface (pointed nodules, 2.5 mm apart)  C. textured surface (circular nodules, 5 mm apart)  [unshod, insoles; 3 mm thick, shore A50] | Upright balance (double); 30 s:  i. EO, EC | Postural sway (CoP excursion); force plate:  1. AP length  2. AP SD  3. ML length  4. ML SD (mm)  5. Velocity (mm/s)  EMG; surface (BF, MG, RF, TA, VL):  6. Amplitude (µV) | 3 and 4 decreased^f^ in EC condition (vs. A)  [interaction tests nr] |
| (Hatton et al., 2012) [9] [RM] – conference proceeding | 26 fallers, 19 F; 79.0±7.1  Note: ≥2 falls previous yr | Plantar surface ((in)direct nr); textured insole (uniformity nr):  A. control insole  B. textured insole (nr)  [participant’s shoes] | Walking (self preferred speed); 10 m | Gait (lower limb: spatiotemporal); instrumented surface:  1. BoS (cm)  2. Cadence (steps/min)  3. Support time (step cycle, swing, stance, single and double limb support (s))  4. Step length  5. Stride length (cm)  6. Walking speed (cm/s) | 5 and 6 decreased^f^ (vs. A)  Note: reduction interpreted as a more cautious |
| *(Jenkins et al., 2009) [10] [MM] | 80: healthy age-matched subgroup (n = 40, 25 F); 64.73±7.66, Parkinson’s disease subgroup (n = 40, 24 M); 65.40±8.01 | Planter surface ((in)direct nr); textured insole (uniform):  A. control insole  B. textured insole (raised ridge, ~2 mm height around perimeter of otherwise flat insole)  [shoes provided] | Walking (self-preferred speed); 6.10 m | Gait (lower limb: spatiotemporal); instrumented surface:  1. Single limb support time (ms)  2. Step length  3. Step to step variability (cm)  4. Walking speed (cm/s)  EMG (n = 20 per subgroup); surface (BF, LG, Q, TA):  5. Amplitude (µV)  6. Time to peak activation (ms)  [phases: HS, single limb, terminal, swing] | No significant main effects of texture (vs. A)  [1 texture x disease x trial interaction^g^]  [6 texture x disease x phase interaction^g^] |
| *(Kelleher et al., 2010) [14] [MM] | 24: healthy age-matched subgroup (n = 10, 8 M); 37.2±7.08, MS subgroup (n = 14, 12 F); 41.8±7.3  Note: MS subgroup tested worse^f^ (vs. healthy subgroup) for planter sensitivity | Plantar surface (direct); textured insole (uniform):  A. control insole  B. textured insole (sandpaper, 0.2 mm particle width, only MS participants tested under textured insole conditions)  [shoes provided] | Walking (self-preferred speed); 21 m | Gait (CoM: spatiotemporal, lower limb: kinematics, kinetics); video 3D analysis, force plate:  1. Cadence (steps/min)  2. CoM velocity (m/s)  3. Hip, knee and ankle (max, min, total excursions during HS and TO (deg))  4. GRF (N/kg) (max and min)  EMG; surface (LG, MG, SOL, TA):  5. Amplitude (µV)  [Phases: HC to peak GRF, to TO, swing] | 3, 4 improved^f^, 5 worsened^f^ (relative to healthy subgroup levels) in the MS subgroup (vs. A) |
| *(Maki et al., 1999) [6]  [MM] | 21 healthy: young subgroup (n = 7, 5 F); 26 (23-31), elderly subgroup (n = 14, 8 M); 69 (65-73) | Planter surface (direct); flexible tubing (uniform):  A. control surface (force plate)  B. textured material (plastic tubing; 3 mm thick, taped 1 cm in from the boundary of the plantar surface)  [unshod]  Note: in the elderly subgroup additional tubing was taped transversely across the MT heads | Upright balance (double, EC);  i. continuous platform perturbation (counted backwards in threes)  ii. intermittent small, large perturbation  [young subgroup instructed; ‘try not to step’, elderly subgroup given no instructions] | Postural sway (CoP excursion, strategy); force plate, video 2D analysis:  1. CoP distance to BoS boundary (%) (forward, back, lateral)  2. Path length with step (%height)  3. Velocity with step (%height/s) (AP, ML),  4. Step length (%height) (AP, ML)  5. Extra steps and arm movements (frequency)  6. Rate of loading of steps (BW/s) | 1 and 5 decreased^f^ in both subgroups (vs. A)  6 decreased^f^ in elderly subgroup (vs. A)  [interaction tests nr] |
| *(McKeon et al., 2012) [15] [RM] | 20 chronic ankle instability, 12 M; 21.5±5.51 | Plantar surface (indirect); textured insole (uniform):  A. control (shod, no insert)  B. sham insole (foam)  C. textured insole (plastic nodules, 4 nodules/cm^2^)  [participant’s shoes, socks provided, insole 3 mm thick] | Upright balance (single); 10 s:  i. EO, EC | Postural sway (CoP excursion); force plate:  1. AP min  2. AP min SD  3. ML min  4. ML min SD (time to boundary (s)) | 3 and 4 worsened^f^ (vs. A and B)  [1 vision x texture interaction^g^] |
| *(Nurse et al., 2005) [21] [RM] | 15 healthy, 12 M; 24.7±2.9 | Plantar surface (direct); textured insole (uniform):  A. control insole (shore C60)  B. textured insole (foam, semicircular nodules 8 mm apart)  [unshod, medical adhesive, stocking and elastic wrap maintained contact between insoles and feet surfaces] | Walking (1.5 m/s required velocity); 30 m | Gait (lower limb: kinematics, kinetics); force plate, video 3D analysis:  1. Knee, ankle and tibia to mid-foot angle (deg) (all planes)  2. Torque peak (knee, ankle (N))  3. GRF impact peak, 1^st^ and 2^nd^ active peaks (N), and time to peak (msec) (AP, ML and vertical)  EMG (lower limb); surface (right leg: BF, LG, MG, RF, SOL, TA, VM):  4. Amplitude intensity (µV^2^) (low, high and average)  [stance phase: 0-20, 20-70, 70-100%] | 1 (frontal tibia to midfoot) and 3 (vertical GRF impact, 1^st^ active and time to peak) increased^f^ (vs. A)  2 (internal knee) and 4 (SOL, TA) decreased^f^ (vs. A) [interactions nr] |
| *(Palluel et al., 2008) [2] [MM] | 38 healthy: young subgroup (n = 19, 10 M); 25.9 (21-32), elderly subgroup (n = 19, 11 F); 69.0 (62-80) | Plantar surface (direct); textured sandal (nonuniform):  A. control insole (3 mm thick, overlaid textured sandal surface)  B. textured sandal (plastic spikes, medial surface; 2 spikes/cm^2^, 1 cm height, 4 mm diameter; remaining surface, 4 spikes/cm^2^, 5 mm height, 3 mm diameter) | Upright balance (double, EC); 32 s:  i. pre-post (5 min period of standing or walking in between)  [control condition always followed textured condition, order of treatment not controlled] | Postural sway (CoP excursion); force plate:  1. Area (mm^2^)  2. AP RMS  3. AP MF  4. MLRMS (mm)  5. ML MF (Hz)  6. Velocity (mm/s) | 1, 2 and 4 decreased^f^ in both groups (vs. A)  2 decreased^f^ in elderly subgroup (vs. A)  [1 and 2 texture x age x resting task x follow up interaction^g^]  [6 texture x age x follow up interaction^g^]  [4 texture x follow up interaction^g^] |
| *(Palluel & Nougier, 2009) [3] [MM] | 36 healthy: young subgroup (n = 17, 10 F); 24.3 (21-32), elderly subgroup (n = 19, 11 F); 68.0 (61-80) | Identical to (Palluel et al., 2008) | Upright balance (double, EC); 32 s:  i. pre-post (5 min period of standing or walking in between tests (textured sandals on) followed by pre-post with 5 min of sitting in between (control insole only))  [control condition always followed textured condition, order of treatment not controlled] | Postural sway (CoP excursion); force plate:  1. Area (mm^2^)  2. AP RMS  3. ML RMS (mm)  4. Velocity (mm/s) | 1 and 2 decreased^f^ in both subgroups (vs. A)  [1 and 2 texture x age x resting task interaction^g^] |
| *(Perry et al., 2008) [11] [MM] | 40 moderate insensitivity: control insole trained subgroup (n = 20, 10 F); 69±3.1, textured insole trained subgroup (n = 20, 11 M); 69±3.6 | Planter surface ((in)direct nr); textured insole (uniform):  A. control insole  B. textured insole (raised ridge, ~2 mm height around perimeter of otherwise flat insole)  [shoes provided] | Walking (self-preferred speed, uneven surface); 8 m:  i. pre-post (12 weeks self-regulated activity in between tests)  [no significant difference^f^ between group activity logs] | Gait (CoM and lower limb: kinematics and kinetics); force plate, video 3D analysis:  1. CoM-BoS min lateral distance (cm)  [stance phase] | 1 increased^f^ (vs. A)  [no significant interactions]  Note: no change with 12 weeks of wearing for both subgroups. 14 participants fell during 12 week period, 9 of which were assigned to the control insole trained group |
| *(Preszner-Domjan et al., 2012) [4] [RM] | 50 healthy, 34 F; 23±2 | Plantar surface (direct); textured surface (uniform):  A. control surface (force plate)  B. textured surface (density 5 spikes/cm^2^, 2 mm diameter, 7 mm height)  C. massage | Upright balance (double); 10 s:  i. EO, EC  ii. hard, foam | Postural sway (CoP excursion); force plate:  1. AP length  2. ML length (mm) | 1 and 2 decreased^f^ (vs. A) on hard surface only  [1 and 2 texture x vision interaction^g^ on hard surface] |
| *(Qiu et al., 2012) [5] [MM] | 17 healthy: young subgroup (n = 10, 6 M); 27±3, elderly subgroup (n = 7, 4 M); 72±4 | Plantar surface (direct); textured surface (uniform):  A. control surface (force plate)  B. textured surface (hard: 320 density)  C. textured surface (soft: 270 density)  [unshod, nodules, 5 mm diameters, 3.1 mm heights, 1.5 mm thicknesses] | Upright balance (double); 30 s:  i. EO, EC  ii. hard, foam | Postural sway (CoP excursion); force plate:  1. Area (mm^2^)  2. AP length  3. ML length  4. Path length (mm) | 1, 2, 3 and 4 decreased^f^ in elderly subgroup (vs. A)  2 and 3 decreased in young subgroup (vs. A)  [1, 2, 3 and 4 texture x age x surface  Interaction^g^] |
| (Ritchie et al., 2011) [22] [RM] – Data not suitable | 21 healthy M: 21.0±4.0 | Plantar surface (direct); textured insole (uniform):  A. control insole  B. orthotic insole (designed to reduce pronation)  C. textured insole (hard plastic nodules, 12 mm apart, 4 mm diameter, inner medial third only)  [shoes provided] | Walking (self-preferred speed); 15 metre | Gait (lower limb: kinematics); video 3D analysis:  1. Peak frontal mid-foot-tibia angle (deg)  [phases: loading, midstance, propulsion]  EMG (lower limb); surface (MG, PL, TA):  2. Average intensity (mV)  [phases: pre HC, loading, midstance, propulsion] | 1 increased^f^ (vs. A and B)  [interactions nr] |
| *(Tremblay et al., 2004) [12] [MM] | 61 healthy: young subgroup (n = 25, 13 M); 23±3, elderly subgroup (n = 36, 26 F); 70±8 | Fingertip (direct); textured surface (uniform):  A. no contact  B. smooth surface (inter-ridge distance 0.7 mm, ridge width 0.2 mm, groove width 0.5)  C. textured surface (inter-ridge distance 2.7 mm, ridge width 0.2 mm, groove width 2.5 mm) | Upright balance (double); 60 s:  i. EO, EC  ii. hard, foam | Postural sway (CoP excursion); force plate:  1. AP length  2. ML length (cm)  Fingertip contact force (right index); strain gauge:  3. Force (N) | No significant findings (texture vs. smooth)  [no significant interaction effects] |
| *(Waddington & Adams, 2000) [23] [RM] | 12 athlete F; 18±0.9 | Plantar surface (indirect); textured insole (uniform):  A. barefoot  B. socks  C. shoes and socks  D. shoes, socks and textured insole (rubber nodules, 4 per cm^2^, 7mm height)  [shoe controls nr, socks provided] | Verbalized ankle inversion movement discrimination task:  i. pre-mid-post (wobble board training with 18 weeks between sessions)  [pre session; A and B tested, mid session; A and C tested, post session; A and D tested. Order of treatment not controlled] | Psychophysical test (inversion platform); method of absolute judgment:  1. Descriminability [five positions relative to horizontal (deg) tested: 10.49, 11.84, 12.55, 13.27, 14.52] | 1 increased^f^ (vs. C) |
| *(Waddington & Adams, 2003) [24] [RM] | 17 athlete F; 24±5 | Plantar surface (indirect); textured insole (uniform):  A. barefoot  B. shoes and socks  C. shoes, socks, and textured insoles (rubber nodules, 4 per cm^2^, 7 mm height)  [shoe controls nr, sock control nr] | Verbalized ankle inversion movement discrimination task | Psychophysical test (inversion platform); method of absolute judgment:  1. Descriminability [five positions away from horizontal tested] | 1 increased^f^ (vs. B) |
| *(Watanabe & Okubo, 1981) [1] [MM] | 20 healthy M; 24.4 | Plantar surface (direct); textured surface (uniform):  A. control surface (force plate)  B. textured surface (1 cm apart)  C. textured surface (1.5 cm apart)  D. textured surface (2 cm apart)  [pellets, 1 mm height] | Upright balance (double); 20 s:  i. EO, EC (n = 16)  ii. normal breathing, held with EC (n = 10)  [order of treatment nr] | Postural sway (CoP excursion); force plate:  1. Area (mm^2^)  2. AP length  3. ML length  4. Path length (mm)  Nerve discharge (n = 2, Hoffmann’s reflex); needle electrodes:  5. Hoffmann and M-wave amplitude (µV) (tibial nerve) | 1 and 4 decreased^f^ (B, C and D vs. A)  [interaction nr]  Note: the more densely packed the nodules per unit area, the stronger the effect was on reducing postural sway |
| (Wilson et al., 2008) [18] [MM] – Data not suitable | 40 healthy F; 51.1±5.8 | Plantar surface (indirect); textured insole (uniform):  A. control insole (shore value A20)  B. orthotic insole  C. textured orthotic insole (circular nodules)  D. textured orthotic insole (pointed nodules)  [shoes provided, socks provided, insoles 3 mm thick] | Upright balance (double); 30 s:  i. EO, EC  Walking (self-preferred speed): 7.6 m:  ii. pre-post (4 weeks of self-regulated activity, participants had a required minimum wear time) | Postural sway (CoP excursion); force plate:  1. AP length  2. ML length (cm)  Gait (lower limb: spatiotemporal); instrumented walkway:  3. BoS (cm) | No significant findings |
| Footnotes  a Data for ages are presented in whole y, means, means ± SDs or SEs, and ranges or not reported where stated.  b Information for the intervention are presented or not reported where stated, including: body region, absence or presence of separating materials; textured characteristics and control material characteristics, and; controls. Each material used lettered A to D throughout the studies.  c Information for task characteristics during measurement are presented, including the primary task and task manipulations. Each manipulation was given roman numerals i to ii throughout the studies.  d Information for the different outcomes are presented, including: measurement type and data type, and; instrument used. Different outcomes are numbered 1 to 6 throughout the studies.  e Summary of the significant effects of texture during the performance measure.  f p<0.05 vs control condition or group (control conditions/groups refer to no texture conditions/groups)  g p<0.05 interaction effect (only those involving texture are reported)  Abbreviation index: º = degrees; * = studies meet inclusion criteria in the meta-analysis, un-stared studies meet primary but not secondary criteria; **µV** = millivolts; **AP** = anterior-posterior; **BF** = biceps femoris; **BoS** = base of support; **BW** = body weight; **cm** = centimeters; **cm/s** = centimeters per second; **CoM** = centre of mass; **CoP** = centre of pressure; **DF** = ankle dorsi flexion; **EO** = eyes open; **EC** = eyes closed; **EMG** = electromyography; **F** = female; **GRF** = ground reaction force; **HS** = heel strike; **Hz** = cycles/second; **int** = internal; **KE** = knee extension; **KF** = knee flexion; **km/hr** = kilometers per hour; **LG** = lateral gastrocnemius; **M** = male; **m** = metres; **MF** = median frequency; **MG** = medial gastrocnemius; **min** = minutes; **ML** = medial-lateral; **mm** = millimeters; **MM** = mixed model ANOVA design; **MP** = mastoid process; **ms** = milliseconds; **MS** = multiple sclerosis; **m/s** = metres per second; **MT** = metatarsal; **N** = newton’s; **N/kg** = Newton’s per kilogram; **OC** = occipital protuberances; **PF** = ankle plantar flexion; **PL** = peroneus longus; **Q** = quadriceps; **rep/min** = repetions per minute; **RF** = rectus femoris; **s** = seconds; **RM** = repeated measures ANOVA design; **RMS** = root mean square; **SC** = steroclavicular; **SCM** = sternocliedomastoid; **SD** = standard deviation; **SOL** = soleus; **TA** = tibialis anterior; **TO** = toe-off; **TP** = trapezius; **VL** = vastus lateralis; **VM** = vastus medialis; **W** = watts. | | | | | |
